# Supplementary material for: Multi‐dataset identification of innovative feature genes and molecular mechanisms in keratoconus
Source: J Cell Mol Med. 2024 Sep 19;28(18):e70079. doi: 10.1111/jcmm.70079 (PMC11412914; doi:10.1111/jcmm.70079)
Supplement: Supplementary file 2 — Material S2. [file JCMM-28-e70079-s002.zip › 7.GSEA analysis/2.ATOH7/KEGG_ALZHEIMERS_DISEASE.html]

Details for gene set KEGG\_ALZHEIMERS\_DISEASE[GSEA]

|  || Dataset | ATOH7.group.cls#h\_versus\_l |
| Phenotype | group.cls#h\_versus\_l |
| Upregulated in class | h |
| GeneSet | KEGG\_ALZHEIMERS\_DISEASE |
| Enrichment Score (ES) | 0.33202025 |
| Normalized Enrichment Score (NES) | 1.7230082 |
| Nominal p-value | 0.007874016 |
| FDR q-value | 0.24287102 |
| FWER p-Value | 0.33 |
Table: GSEA Results Summary

  

Fig 1: Enrichment plot: KEGG\_ALZHEIMERS\_DISEASE      
 Profile of the Running ES Score & Positions of GeneSet Members on the Rank Ordered List

  

| SYMBOL | TITLE | RANK IN GENE LIST | RANK METRIC SCORE | RUNNING ES | CORE ENRICHMENT || 1 | NOS1 | na | 0 | 1.649 | 0.1388 | Yes |
| 2 | SNCA | na | 94 | 0.777 | 0.2015 | Yes |
| 3 | APBB1 | na | 879 | 0.466 | 0.2180 | Yes |
| 4 | COX8C | na | 1278 | 0.411 | 0.2411 | Yes |
| 5 | PLCB1 | na | 1718 | 0.367 | 0.2593 | Yes |
| 6 | MAPT | na | 1744 | 0.365 | 0.2893 | Yes |
| 7 | MME | na | 2632 | 0.301 | 0.2890 | Yes |
| 8 | APOE | na | 2761 | 0.295 | 0.3101 | Yes |
| 9 | GRIN2C | na | 3488 | 0.256 | 0.3107 | Yes |
| 10 | CALML6 | na | 3497 | 0.256 | 0.3320 | Yes |
| 11 | IL1B | na | 6104 | 0.168 | 0.2708 | No |
| 12 | PPP3CC | na | 7238 | 0.137 | 0.2496 | No |
| 13 | FAS | na | 7283 | 0.136 | 0.2598 | No |
| 14 | BACE1 | na | 7392 | 0.133 | 0.2679 | No |
| 15 | ITPR1 | na | 7473 | 0.131 | 0.2766 | No |
| 16 | APAF1 | na | 7669 | 0.126 | 0.2816 | No |
| 17 | NDUFA4L2 | na | 8390 | 0.110 | 0.2700 | No |
| 18 | CACNA1S | na | 8751 | 0.103 | 0.2683 | No |
| 19 | GRIN1 | na | 8945 | 0.100 | 0.2712 | No |
| 20 | CALM3 | na | 10070 | 0.080 | 0.2454 | No |
| 21 | ADAM17 | na | 10662 | 0.071 | 0.2343 | No |
| 22 | EIF2AK3 | na | 10954 | 0.067 | 0.2315 | No |
| 23 | APH1A | na | 11156 | 0.064 | 0.2310 | No |
| 24 | CYC1 | na | 11419 | 0.060 | 0.2285 | No |
| 25 | ATF6 | na | 11511 | 0.059 | 0.2308 | No |
| 26 | PPP3CA | na | 11626 | 0.057 | 0.2324 | No |
| 27 | ADAM10 | na | 11689 | 0.057 | 0.2353 | No |
| 28 | SDHB | na | 11751 | 0.056 | 0.2383 | No |
| 29 | TNFRSF1A | na | 11768 | 0.055 | 0.2425 | No |
| 30 | CDK5R1 | na | 11797 | 0.055 | 0.2463 | No |
| 31 | PPP3CB | na | 11848 | 0.055 | 0.2494 | No |
| 32 | CDK5 | na | 12266 | 0.049 | 0.2415 | No |
| 33 | HSD17B10 | na | 12512 | 0.046 | 0.2383 | No |
| 34 | COX7B2 | na | 12668 | 0.044 | 0.2375 | No |
| 35 | UQCRB | na | 13482 | 0.035 | 0.2170 | No |
| 36 | NDUFS2 | na | 13540 | 0.035 | 0.2183 | No |
| 37 | ATP2A3 | na | 13569 | 0.035 | 0.2204 | No |
| 38 | NDUFA6 | na | 13652 | 0.034 | 0.2209 | No |
| 39 | UQCRH | na | 13770 | 0.033 | 0.2203 | No |
| 40 | COX7A1 | na | 14036 | 0.030 | 0.2151 | No |
| 41 | CASP9 | na | 14196 | 0.029 | 0.2130 | No |
| 42 | NDUFV2 | na | 14197 | 0.029 | 0.2154 | No |
| 43 | COX4I1 | na | 14252 | 0.028 | 0.2161 | No |
| 44 | NAE1 | na | 14271 | 0.028 | 0.2180 | No |
| 45 | TNF | na | 14399 | 0.027 | 0.2165 | No |
| 46 | NDUFA2 | na | 14444 | 0.026 | 0.2175 | No |
| 47 | PSENEN | na | 14523 | 0.026 | 0.2174 | No |
| 48 | MAPK1 | na | 14536 | 0.025 | 0.2192 | No |
| 49 | CHP1 | na | 14674 | 0.024 | 0.2172 | No |
| 50 | GAPDH | na | 14852 | 0.022 | 0.2140 | No |
| 51 | NDUFA4 | na | 14988 | 0.021 | 0.2119 | No |
| 52 | UQCRFS1 | na | 15042 | 0.021 | 0.2121 | No |
| 53 | COX6A1 | na | 15045 | 0.021 | 0.2138 | No |
| 54 | NDUFA7 | na | 15138 | 0.020 | 0.2128 | No |
| 55 | COX7A2L | na | 15220 | 0.019 | 0.2121 | No |
| 56 | NDUFB7 | na | 15235 | 0.019 | 0.2133 | No |
| 57 | NDUFA3 | na | 15254 | 0.019 | 0.2143 | No |
| 58 | COX4I2 | na | 15306 | 0.018 | 0.2144 | No |
| 59 | PPP3R1 | na | 15411 | 0.018 | 0.2129 | No |
| 60 | SDHA | na | 15471 | 0.017 | 0.2126 | No |
| 61 | NDUFS7 | na | 15694 | 0.015 | 0.2075 | No |
| 62 | SDHD | na | 15796 | 0.014 | 0.2058 | No |
| 63 | COX5A | na | 15943 | 0.013 | 0.2027 | No |
| 64 | GSK3B | na | 16028 | 0.013 | 0.2013 | No |
| 65 | UQCR10 | na | 16088 | 0.012 | 0.2006 | No |
| 66 | UQCRC2 | na | 16211 | 0.011 | 0.1980 | No |
| 67 | CALM1 | na | 16251 | 0.011 | 0.1978 | No |
| 68 | CYCS | na | 16260 | 0.011 | 0.1985 | No |
| 69 | NDUFA9 | na | 16374 | 0.010 | 0.1960 | No |
| 70 | COX7C | na | 16469 | 0.009 | 0.1941 | No |
| 71 | NDUFB4 | na | 16810 | 0.006 | 0.1848 | No |
| 72 | MAPK3 | na | 16821 | 0.006 | 0.1850 | No |
| 73 | UQCRQ | na | 16876 | 0.006 | 0.1839 | No |
| 74 | NDUFB2 | na | 17205 | 0.003 | 0.1746 | No |
| 75 | COX8A | na | 17213 | 0.003 | 0.1747 | No |
| 76 | BAD | na | 17218 | 0.002 | 0.1747 | No |
| 77 | NDUFB6 | na | 17476 | 0.000 | 0.1673 | No |
| 78 | NDUFB3 | na | 17733 | -0.002 | 0.1601 | No |
| 79 | NDUFB9 | na | 17840 | -0.003 | 0.1572 | No |
| 80 | NDUFA5 | na | 17888 | -0.003 | 0.1561 | No |
| 81 | NDUFC1 | na | 17903 | -0.003 | 0.1560 | No |
| 82 | COX6C | na | 17968 | -0.004 | 0.1545 | No |
| 83 | NDUFS8 | na | 18032 | -0.004 | 0.1530 | No |
| 84 | COX7A2 | na | 18090 | -0.005 | 0.1518 | No |
| 85 | CAPN2 | na | 18157 | -0.005 | 0.1503 | No |
| 86 | NDUFB10 | na | 18344 | -0.007 | 0.1455 | No |
| 87 | UQCRC1 | na | 18411 | -0.008 | 0.1443 | No |
| 88 | CAPN1 | na | 18438 | -0.008 | 0.1442 | No |
| 89 | NDUFA8 | na | 18467 | -0.008 | 0.1440 | No |
| 90 | NDUFS3 | na | 18481 | -0.008 | 0.1444 | No |
| 91 | COX5B | na | 18689 | -0.010 | 0.1392 | No |
| 92 | ITPR3 | na | 18764 | -0.011 | 0.1380 | No |
| 93 | NDUFB8 | na | 18767 | -0.011 | 0.1389 | No |
| 94 | NDUFS4 | na | 18768 | -0.011 | 0.1398 | No |
| 95 | COX6B1 | na | 19017 | -0.013 | 0.1337 | No |
| 96 | CASP7 | na | 19147 | -0.014 | 0.1312 | No |
| 97 | NDUFAB1 | na | 19284 | -0.015 | 0.1285 | No |
| 98 | SDHC | na | 19318 | -0.016 | 0.1289 | No |
| 99 | COX7B | na | 19335 | -0.016 | 0.1297 | No |
| 100 | UQCR11 | na | 19468 | -0.017 | 0.1273 | No |
| 101 | NDUFV3 | na | 19559 | -0.018 | 0.1262 | No |
| 102 | LPL | na | 19576 | -0.018 | 0.1273 | No |
| 103 | NDUFS5 | na | 19842 | -0.020 | 0.1213 | No |
| 104 | NDUFA1 | na | 19847 | -0.020 | 0.1229 | No |
| 105 | BACE2 | na | 19858 | -0.020 | 0.1243 | No |
| 106 | NDUFV1 | na | 19894 | -0.021 | 0.1250 | No |
| 107 | NDUFC2 | na | 19991 | -0.022 | 0.1241 | No |
| 108 | NDUFB1 | na | 20064 | -0.022 | 0.1239 | No |
| 109 | IDE | na | 20190 | -0.024 | 0.1223 | No |
| 110 | NCSTN | na | 20251 | -0.024 | 0.1226 | No |
| 111 | PSEN2 | na | 20477 | -0.027 | 0.1183 | No |
| 112 | BID | na | 20508 | -0.027 | 0.1197 | No |
| 113 | CALML3 | na | 20596 | -0.028 | 0.1195 | No |
| 114 | GNAQ | na | 20613 | -0.028 | 0.1214 | No |
| 115 | PLCB4 | na | 20753 | -0.029 | 0.1198 | No |
| 116 | NDUFS6 | na | 21061 | -0.032 | 0.1136 | No |
| 117 | ERN1 | na | 21531 | -0.038 | 0.1032 | No |
| 118 | FADD | na | 21903 | -0.042 | 0.0960 | No |
| 119 | CALM2 | na | 21949 | -0.042 | 0.0983 | No |
| 120 | CACNA1C | na | 22079 | -0.044 | 0.0982 | No |
| 121 | NDUFB5 | na | 22347 | -0.047 | 0.0945 | No |
| 122 | LRP1 | na | 22679 | -0.051 | 0.0891 | No |
| 123 | NDUFS1 | na | 22850 | -0.053 | 0.0887 | No |
| 124 | APP | na | 22972 | -0.054 | 0.0897 | No |
| 125 | PSEN1 | na | 22990 | -0.055 | 0.0938 | No |
| 126 | NDUFA10 | na | 23223 | -0.058 | 0.0920 | No |
| 127 | CASP8 | na | 23310 | -0.059 | 0.0945 | No |
| 128 | ATP2A2 | na | 23505 | -0.062 | 0.0941 | No |
| 129 | PLCB3 | na | 24121 | -0.071 | 0.0823 | No |
| 130 | COX6B2 | na | 24380 | -0.075 | 0.0812 | No |
| 131 | CASP3 | na | 24828 | -0.083 | 0.0752 | No |
| 132 | GRIN2D | na | 25022 | -0.086 | 0.0769 | No |
| 133 | CACNA1D | na | 26374 | -0.112 | 0.0472 | No |
| 134 | ITPR2 | na | 26843 | -0.122 | 0.0440 | No |
| 135 | CACNA1F | na | 27070 | -0.127 | 0.0481 | No |
| 136 | GRIN2B | na | 28274 | -0.158 | 0.0267 | No |
| 137 | PLCB2 | na | 28336 | -0.160 | 0.0384 | No |
| 138 | GRIN2A | na | 28440 | -0.163 | 0.0491 | No |
| 139 | CHP2 | na | 28497 | -0.164 | 0.0613 | No |
| 140 | ATP2A1 | na | 29423 | -0.192 | 0.0507 | No |
| 141 | CALML5 | na | 29931 | -0.208 | 0.0536 | No |
| 142 | COX6A2 | na | 30724 | -0.237 | 0.0506 | No |
| 143 | PPP3R2 | na | 32636 | -0.337 | 0.0237 | No |
| 144 | RYR3 | na | 33675 | -0.436 | 0.0304 | No |
Table: GSEA details [plain text format]

  

Fig 2: KEGG\_ALZHEIMERS\_DISEASE      
 Blue-Pink O' Gram in the Space of the Analyzed GeneSet

  

Fig 3: KEGG\_ALZHEIMERS\_DISEASE: Random ES distribution      
 Gene set null distribution of ES for **KEGG\_ALZHEIMERS\_DISEASE**
